# Bisacurone suppresses hepatic lipid accumulation through inhibiting lipogenesis and promoting lipolysis

**DOI:** 10.3164/jcbn.20-16

**Published:** 2020-05-08

**Authors:** Hitoshi Ashida, Xiaokuo Tian, Tomoya Kitakaze, Yoko Yamashita

**Affiliations:** 1 Department of Agrobioscience, Graduate School of Agricultural Science, Kobe University, 1-1, Rokkodai-cho, Nada-ku, Kobe 657-8501, Japan

**Keywords:** bisacurone, turmeric, lipid metabolism, AMP-activated protein kinase, liver

## Abstract

Turmeric and its components have various health beneficial functions. However, little is known about function of bisacurone, which is one of the sesquiterpenes in turmeric, at the compound level. In this study, we investigated the preventive effect of bisacurone on hepatic lipid accumulation and its mechanism in HepG2 cells and ICR mice. In HepG2 cells, bisacurone significantly inhibited fatty acid-induced intracellular lipid accumulation in a dose-dependent manner. Bisacurone at 10 µM increased protein expression of peroxisome proliferator-activated receptor α and carnitine palmitoyltransferase-1A accompanied by phosphorylation of AMP-activated protein kinase. In the liver of ICR mice, bisacurone decreased total lipids, triglyceride, and cholesterol contents. Bisacurone at 10 mg/kg body weight increased phosphorylation of AMP-activated protein kinase, and its downstream acetyl-CoA carboxylase as a rate-limiting enzyme for lipogenesis, while it decreased the nuclear translocation level of sterol regulatory element-binding protein 1 and carbohydrate-responsive element-binding protein as the major transcription factors for lipogenesis. On the other hand, bisacurone promoted lipolysis by up-expression of peroxisome proliferator-activated receptor α and carnitine palmitoyltransferase-1A. Thus, bisacurone might be a valuable food factor for preventing hepatic lipid accumulation by inhibiting lipogenesis and promoting lipolysis through phosphorylation of AMP-activated protein kinase.

## Introduction

Excess accumulation of lipids in the liver is currently recognized as the most common cause of chronic liver diseases and a risk factor for other diseases.^(1)^ In the liver, lipid accumulation results from an imbalance between increasing lipogenesis and decreasing lipolysis including fatty acid oxidation.^(2–4)^ This imbalance tilted toward to the lipid accumulation causes hepatic steatosis, hyperlipidemia, cardiovascular disease, obesity, and diabetes.^(5)^ AMP-activated protein kinase (AMPK), a serine/threonine protein kinase, plays a key role in energy homeostasis including hepatic lipid metabolism, serving as a metabolic master switch in response to alterations in cellular energy charge.^(6)^ Recent studies have showed that liver kinase B1 (LKB1), a 50 kDa serine/threonine kinase, is one of the important upstream kinases of AMPK. LKB1 can activate AMPK by phosphorylation at Thr172 in mammalian cells. This activation of AMPK is involved in various pathways such as fatty acid, triglyceride, and cholesterol synthesis as well as catabolic pathways such as lipolysis and fatty acid oxidation.^(7)^ Based on this, AMPK-mediated pathways have emerged as the novel targets for the treatment of obesity and fatty liver.^(8)^

AMPK modulates lipid synthesis, lipolysis, and fatty acid oxidation through phosphorylation of key substrates, acetyl-CoA carboxylase (ACC), and sterol regulatory element binding protein 1 (SREBP-1).^(9)^ Inactivation of ACC by its phosphorylation results in an increase in fatty acid oxidation and suppression of fatty acid synthesis, while phosphorylation of SREBP-1 decreases its nuclear translocation resulting in down regulating the expression of lipogenic enzymes, including ACC and fatty acid synthase (FAS).^(10)^ Transcriptional factors, such as carbohydrate-responsive element-binding protein (ChREBP), peroxisome proliferator-activated receptor (PPAR) α and CCAAT/enhancer binding protein (C/EBP) α also regulate lipolysis and fatty acid oxidation in the liver. ChREBP and C/EBPα induce fatty acid synthesis through the induction of lipogenic genes, such as *FAS* and *ACC*, whereas activation of PPARα increase fatty acid oxidation.^(11–13)^ These transcriptional responses also occur in large part through the activation of AMPK.^(14)^

Turmeric (*Curcuma longa* L.) is a traditional Indian food spice which is also widely used as folk medicine.^(15)^ Curcumin is well-documented active compound in turmeric and possesses various health beneficial functions.^(16–19)^ In addition to curcuminoids, turmeric contains various sesquiterpenes, such as bisacurone, turmerones (ar-, α-, and β-), turmeronol (A and B), and curcumenol.^(20,21)^ It was reported that certain sesquiterpenes also possesses health beneficial functions at compound level experiments.^(22–24)^ However, it has little known about the function of bisacrone at a compound level, though one previous report demonstrates bisacurone inhibits adhesion of inflammatory monocytes and oral cancer cells to human umbilical vein endotherial cells through down-regulation of vascular adhesion molecule-1.^(25)^ An aim of this study is investigation of the reduction of hepatic lipid accumulation by bisacurone at the compound level. First, the authors examined the prevention effect of bisacurone on saturated fatty acids-induced lipid accumulation in HepG2. Next, the authors carried out animal experiment using ICR mice to clarify the effects of oral administrated bisacurone on hepatic lipid accumulation *in vivo*. To clarify the underlying molecular mechanisms, the authors focused on the AMPK-driven pathways and investigated the effects of bisacurone on lipogenesis- and lipolysis-related factors in the liver.

## Materials and Methods

### Reagents

**Fig. 1:**
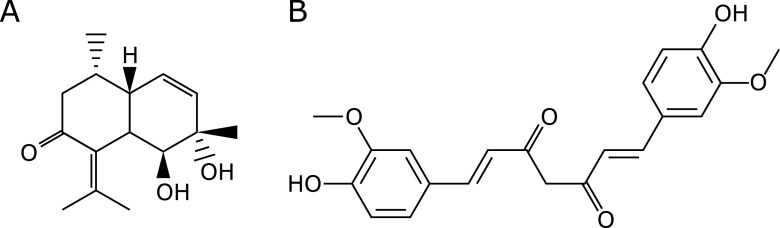
Chemical structures of compounds of (A) bisacurone and (B) curcumin.

Bisacurone was purchased from Nagara Science Co., Ltd. (Gifu, Japan). Curcumin, palmitic acid, oleic acid, and ImmunoStar LD was purchased from FUJIFILM Wako Pure Chemical Industries (Osaka, Japan). Their chemical structures are shown in Fig. 1. Metformin hydrochloride was from Sigma-Aldrich (St. Louis, MO). Dulbecco’s modified Eagle’s medium (DMEM) was obtained from Nissui Pharmaceutical (Tokyo, Japan). Bovine serum albumin (BSA), Blocking One, and Blocking One-P solutions were from Nacalai Tesque, Inc. (Kyoto, Japan). For western blotting, antibodies for lamin B, PPARα, CPT1, fatty acid binding protein 4 (FABP4), C/EBPα, FAS, anti-mouse IgG, anti-goat IgG, and anti-rabbit IgG were purchased from Santa Cruz Biotechnology (Santa Cruz Biotechnology, Santa Cruz, CA); β-actin, p-AMPKα, AMPKα, p-LKB1, and LKB1 were from Cell Signaling Technology (Beverly, MA); ChREBP and SREBP-1 were from Abcam (Cambridge, MA). The polyvinylidene difluoride membrane was products of GE Healthcare Bio-Science Co., (Piscataway, NJ). All other reagents used were of the highest grade available from commercial sources.

### Cell culture and treatments

HepG2 cells were maintained and cultured in Dulbecco’s modified Eagle’s medium (Nissui Pharmaceutical, Tokyo, Japan) containing 10% (v/v) fetal bovine serum (Sigma-Aldrich), 4 mM l-glutamine, 100 U/ml penicillin, and 100 mg/ml streptomycin under a humidified atmosphere of 95% (v/v) air and 5% (v/v) CO_2_ at 37°C. The cells were grown to 80% confluence and incubated in serum-free medium overnight before treatment. The cells were simultaneously treated with 1.2 mM fatty acids mixture (palmitic acid:oleic acid = 1:2 ratio) and various concentrations of bisacurone and curcumin for 24 h. Dimethyl sulfoxide (DMSO) [final concentration is 0.1% (v/v)] was used for a vehicle control. A stock solution of each fatty acid was dissolved in 99% (v/v) methanol and diluted in culture medium containing 1% (w/v) BSA to the final concentration.

### Measurement of cell viability

To determine the cytotoxicity of bisacurone and curcumin against HepG2 cells, crystal violet assay was performed as described previously.^(26)^ Following the treatment with these compounds at 0.01, 0.1, 1, and 10 µM or DMSO as the vehicle control to HepG2 cells in a 96-well plate for 24 h, the cells were washed with PBS, and stained with 2% (v/v) ethanol containing 0.2% (w/v) crystal violet reagent for 10 min in the room temperature. The cells were washed eight times with distilled water to remove excess stain, and the stained cells were solubilized with 50% (v/v) ethanol containing 0.5% (w/v) sodium dodecyl sulfate (SDS). The absorbance was measured at 570 nm using a Wallac 1420 ARVOsx instrument (Perkin-Elmer, Boston, MA). The result of cell viability was expressed as percent of the control cells.

### Determination of intracellular lipid content in HepG2 cells

The inhibitory effect of bisacurone on the lipid accumulation *in vitro*, HepG2 cells were cultured in serum-free medium overnight and then exposed to a mixture of 1.2 mM fatty acids (palmitic acid:oleic acid = 1:2 ratio) with or without 0.01, 0.1, 1.0, and 10 µM of bisacurone for 24 h. Sudan II staining was carried out to determine intracellular lipid accumulation according to the previous report.^(27)^

### Animal experiments

For the animal experiments, thirty-six ICR male mice (5-week-old) mice (Japan SLC, Inc., Shizuoka, Japan) were used to assess the effects of bisacurone on lipid metabolism. Animal experiments were approved by the Kobe University Institutional Animal Care and Use Committee (Permission 28-12-01) and carried out in accordance with the guidelines for animal experiments at Kobe University Animal Experimentation Regulation. Mice were randomly divided to six groups of six each and acclimatized for 7 days with free access to a standard diet (3.850 kcal/g) consisting of 76% (w/w) carbohydrate, 15% (w/w) protein, and 9% (w/w) fat (Research Diets, Tokyo, Japan) and tap water. They were orally administrated with 0.1, 1, or 10 mg/kg body weight (BW) of bisacurone, 10 mg/kg BW of curcumin, 200 mg/kg BW of metformin as a positive control, and polyethylene glycol as a negative control for another 7 consecutive days. Blood, liver, and perirenal, epididymis, mesenteric and subcutaneous white adipose tissues were collected 24 h after the final administration.

### Measurement of hepatic lipid levels

The hepatic lipids were extracted by the method of Folch *et al.*^(28)^ Briefly, an aliquot of approximately 100 mg of liver was homogenized with 0.35 ml of distilled water. Lipids in the homogenates were extracted 3 times with 0.7 ml of a chloroform/methanol (2/1, v/v) solution. After centrifugation at 1,800 × *g* for 10 min, the chloroform layers were collected, and a one-fourth volume of 0.88% (w/v) KCl solution was added. The mixtures were vortexed and centrifuged at 1,800 × *g* for 10 min; the chloroform layers were then collected and evaporated, and the weight of the residue was measured as total lipid. The residue was dissolved in isopropanol containing 10% (v/v) Triton-X and subjected to the measurement of triglyceride and cholesterol levels using corresponding commercially available kit from FUJIFILM Wako Pure Chemical Industries according to the manufacturer’s instructions.

### Measurement of plasma lipids

Blood was centrifuged at 9,600 × *g* for 10 min at 4°C. The supernatant was collected and used as the plasma for measurement of cholesterol and triglyceride levels using the same commercial kits as mentioned above.

### Western blot analysis

Preparation of lysate and the subcellular fractions from HepG2 cells and the liver of ICR mice and western blotting were performed according to our previous report.^(29)^ For the determination of SREBP-1 and ChREBP, proteins were separated by sodium dodecyl sulfate-polyacrylamide gel electrophoresis (SDS-PAGE) using 8% gels. For p-LKB1, LKB1, p-AMPKα, AMPKα, p-ACCα, ACC, PPARα, CPT1, C/EBPα, FAS. FABP4, lamin B, and β-actin, SDS-PAGE was carried out with 10% gels. After SDS-PAGE, the proteins were transferred onto a polyvinylidene fluoride membrane. The membrane was treated with blocking buffer [Blocking One or Blocking One P (for phosphorylated protein)] for 1 h at room temperature. Then, the membranes were incubated with appropriate primary antibody for p-LKB1 (1:5,000), LKB1 (1:10,000), p-AMPKα (1:5,000), AMPKα (1:10,000), p-ACCα (1:5,000), ACCα (1:10,000), PPARα (1:5,000), CPT1 (1:5,000), C/EBPα (1:5,000), FAS (1:5,000), FABP4 (1:5,000), SREBP-1 (1:5,000), ChREBP (1:5,000), lamin B (1:10,000), and β-actin (1:10,000) overnight at 4°C, followed by the corresponding HRP-conjugated secondary antibody (1:10,000) for 2 h at 4°C. The protein bands were visualized using ImmunoStar^®^ LD and specific immune complexes were detected with the ATTO Light-Capture II Western Blotting Detection System (ATTO, Tokyo, Japan). The density of specific band was analyzed using ImageJ analysis software (National Institutes of Health, Bethesda, MD).

### Statistical analysis

The data are expressed as the mean ± SE. Dunnett’s test was used to determine the significance of differences between the treated and control groups. The level of statistical significance was set to *p*<0.05.

## Results

### Effects of turmeric compounds on cell viability in HepG2 cells

**Fig. 2:**
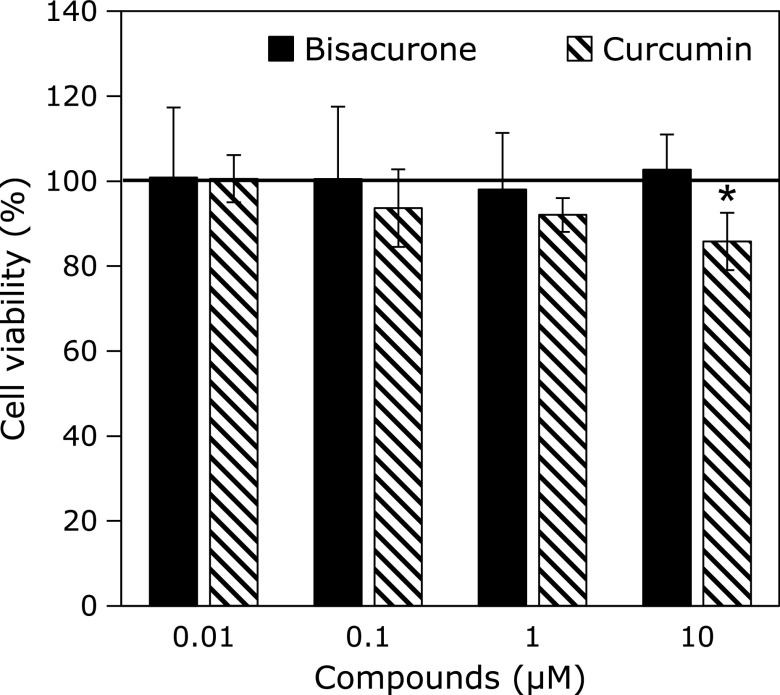
Effects of bisacurone and curcumin on the cell viability in HepG2 cells. The cells were treated to 0.01, 0.1, 1.0, and 10 µM of bisacurone and curcumin for 24 h. DMSO was used as a vehicle control. Cell viability was measured by crystal violet assays. Data are the mean ± SE. Statistical analysis was performed using Dunnett’s test. *p<0.05 vs DMSO-treated control cells.

Cell viability was estimated by the crystal violet staining assay after treatment with indicated concentrations of bisacurone and curcumin for 24 h (Fig. 2). Bisacurone did not show any cytotoxicity by 10 µM, whereas curcumin at 10 µM slightly but significantly exhibited cytotoxicity. α-Curcumene and ar-turmerone, other sesquiterpenes in turmeric, also did not show the cytotoxicity by 10 µM (data not shown). Therefore, bisacurone was treated to the cells at 0.01, 0.1, 1, and 10 µM in the following experiments.

### Bisacurone inhibited fatty acids-induced cellular lipid accumulation in HepG2 cells

**Fig. 3:**
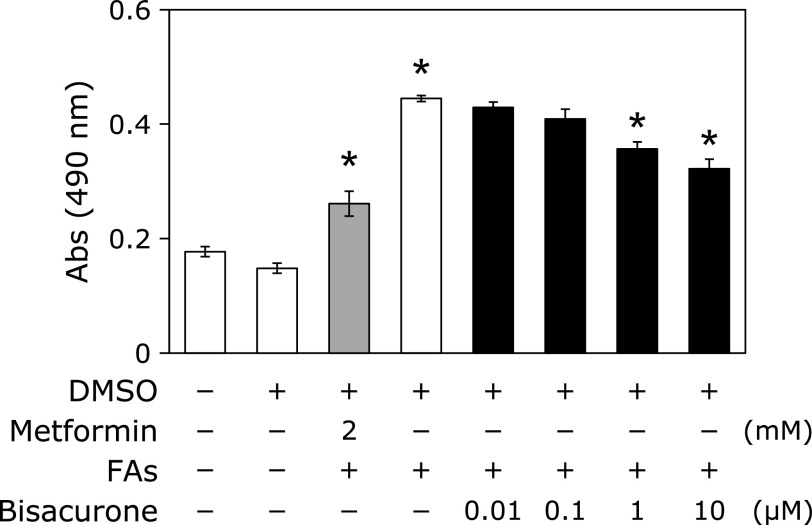
Bisacurone inhibited fatty acids-induced intracellular lipid accumulation in HepG2 cells. The cells were exposed to 1.2 mM fatty acids mixture consisting of 0.4 mM palmitic acid and 0.8 mM oleic acid with or without 0.01, 0.1, 1.0, and 10 µM of bisacurone for 24 h. Metformin (2 mM) and DMSO were used as the positive and negative controls, respectively. Intracellular lipids in the cells were stained with Sudan II and the absorbances of pigment was measured at a wavelength of 490 nm after the pigments were dissolved in isopropanol containing 4% (v/v) Nonidet P-40. Data are the mean ± SE. Statistical analysis was performed using Dunnett’s test. *p<0.05 vs DMSO-treated control cells.

In our previous study, the mixtures of 1:2 proportions of palmitic acid and oleic acid significantly increased lipid accumulation in HepG2 cells.^(27)^ Under the same experimental conditions, we examined whether bisacurone inhibited fatty acids-induced cellular lipid accumulation in HepG2 cells. As shown in Fig. 3, the result from Sudan II staining showed that the mixtures of saturated fatty acids significantly increased lipid accumulation and metformin (2.0 mM) as a positive control significantly reduced lipid accumulation expectedly. Treatment with bisacurone inhibited fatty acids-induced lipid accumulation in a dose-dependent manner, and significant inhibition was observed at 1.0 and 10 µM.

### Effects of bisacurone on lipid metabolism in HepG2 cells

**Fig. 4:**
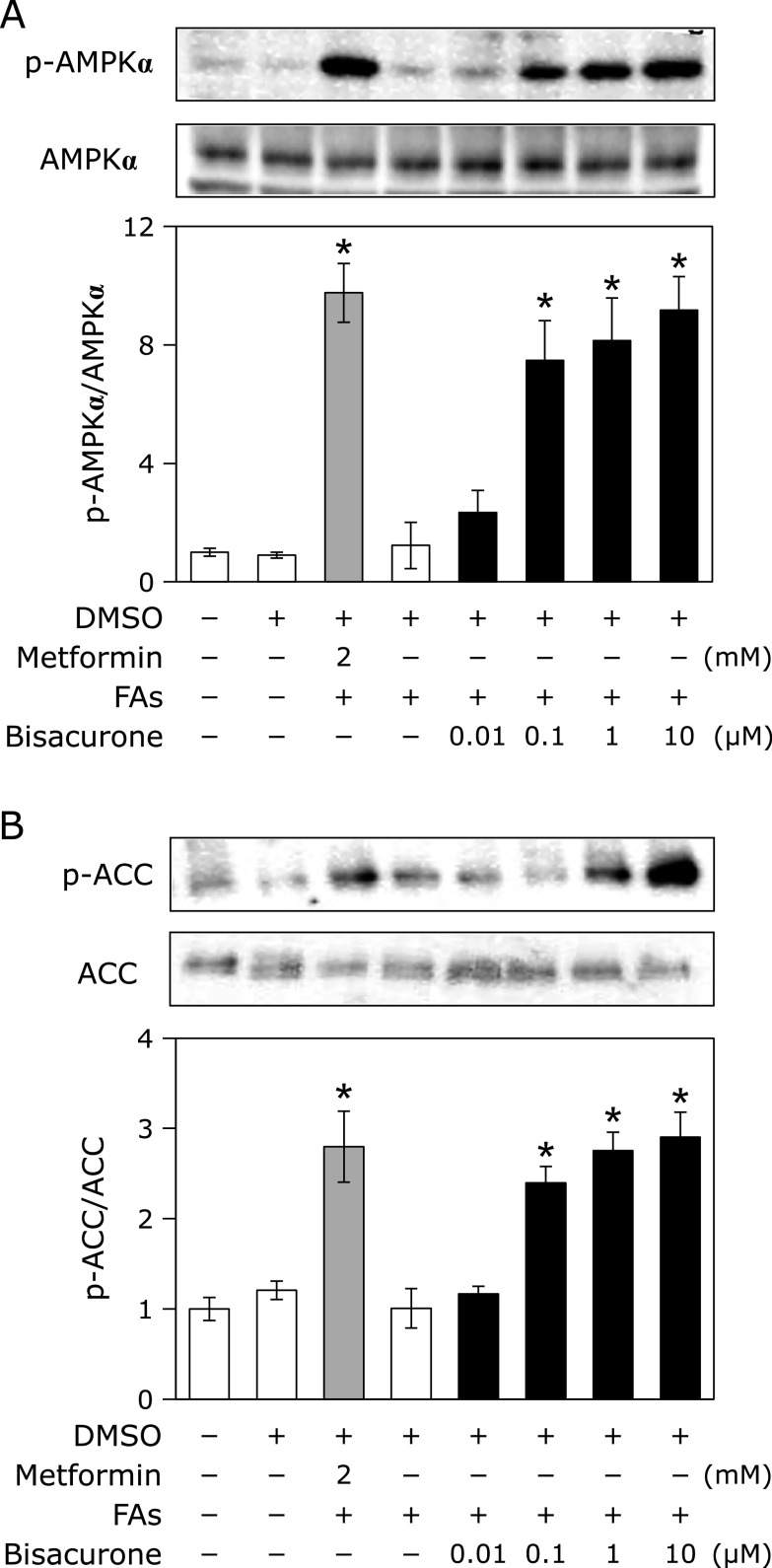
Effects of bisacurone on phosphorylation of AMPKα and ACCα in HepG2 cells. Cell treatment is the same as shown in Fig. 3. The phosphorylation and expression levels of AMPKα and ACCα were determined by western blotting. A typical representative result was shown from the three independent experiments. Density of specific band for phosphorylated protein was normalized by that of corresponding protein expression. Data are the mean ± SE. Statistical analysis was performed using Dunnett’s test. *p<0.05 vs DMSO-treated control cells.

Since AMPK plays a key role in energy homeostasis including hepatic lipid metabolism, AMPK related action was investigated in HepG2 cells after co-treatment with bisacurone and fatty acids. Bisacurone and metformin significantly promoted phosphorylation of AMPKα (bisacurone at 0.1 µM; 7.82 fold, at 1.0 µM; 8.79 fold, at 10 µM; 9.64 fold, and metformin at 2.0 mM; 9.97 fold) in the presence of fatty acids (Fig. 4A). In the absence of fatty acids, both bisacurone and metformin also promoted phosphorylation of AMPKα (data not shown). ACCα is known as a downstream target of AMPKα and a rate-limiting enzyme of lipogenesis.^(9)^ As shown in Fig. 4B, both bisacurone and metformin increased phosphorylation of ACCα (bisacurone at 1.0 µM; 2.75 fold, 10 µM; 2.90 fold, and metformin 2 mM; 2.80 fold). These results indicated that bisacurone inhibited lipogenesis through phosphorylation of ACCα.

**Fig. 5:**
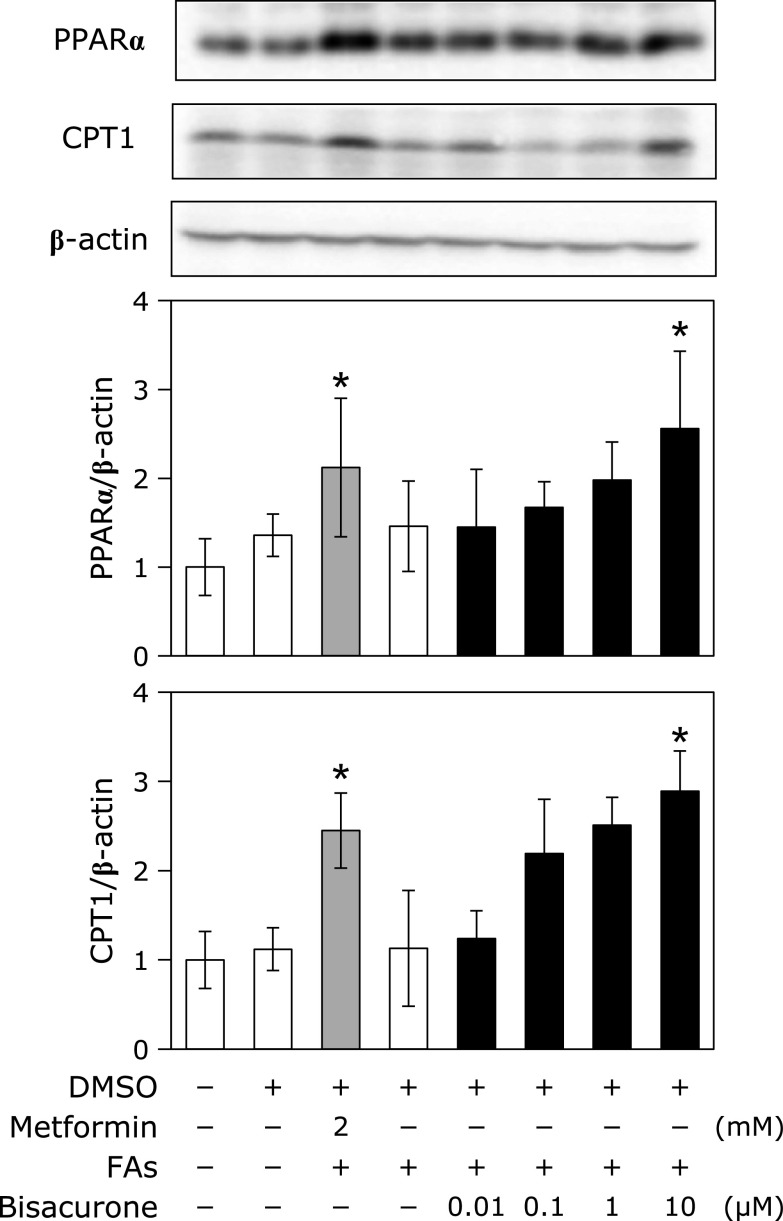
Effects of bisacurone on PPARα and CPT1 expression in HepG2 cells. Cell treatment is the same as shown in Fig. 3. The protein expression level of PPARα and CPT1 was determined by western blotting. A typical representative result was shown from three independent experiments. Density of specific band was normalized by that of β-actin expression. Data are the mean ± SE. Statistical analysis was performed using Dunnett’s test. *p<0.05 vs DMSO-treated control cells.

As to the lipolytic pathway, we focused on two important factors, PPARα and CPT1, which are involved in hepatic fatty acid oxidation and lipolysis, respectively.^(13)^ As shown in Fig. 5, bisacurone increased expression of PPARα (Fig. 5A) and CPT1 (Fig. 5B) in a dose-dependent manner, and the significant increase was observed at 10 µM. It was confirmed that the fatty acids mixture alone did not affect the expression of PPARα and CPT1. These results indicate that bisacurone increase lipolysis through promotion of fatty acids oxidation.

### Changes in the body and tissue weights and plasma lipids of mice after oral administration of bisacurone for 7 days

Next, we explored the *in vivo* experiment to investigate the effects of bisacurone on hepatic lipid metabolism. Orally administered bisacurone for 7 consecutive days did not affect body and liver weight in all experimental groups (Table 1). Weight of white and brown adipose tissues did not alter in all groups, except mesenteric fat of the mice given bisacurone at 1 mg/kg BW. Moreover, there was no change in the levels of plasma triglyceride and total cholesterol (data not shown). These results suggested that an intake of bisacrone did not show the toxicity to the mice under our experimental conditions.

### Oral administration of bisacurone decreased total lipid content, triglyceride, and total cholesterol in the liver of mice

**Fig. 6:**
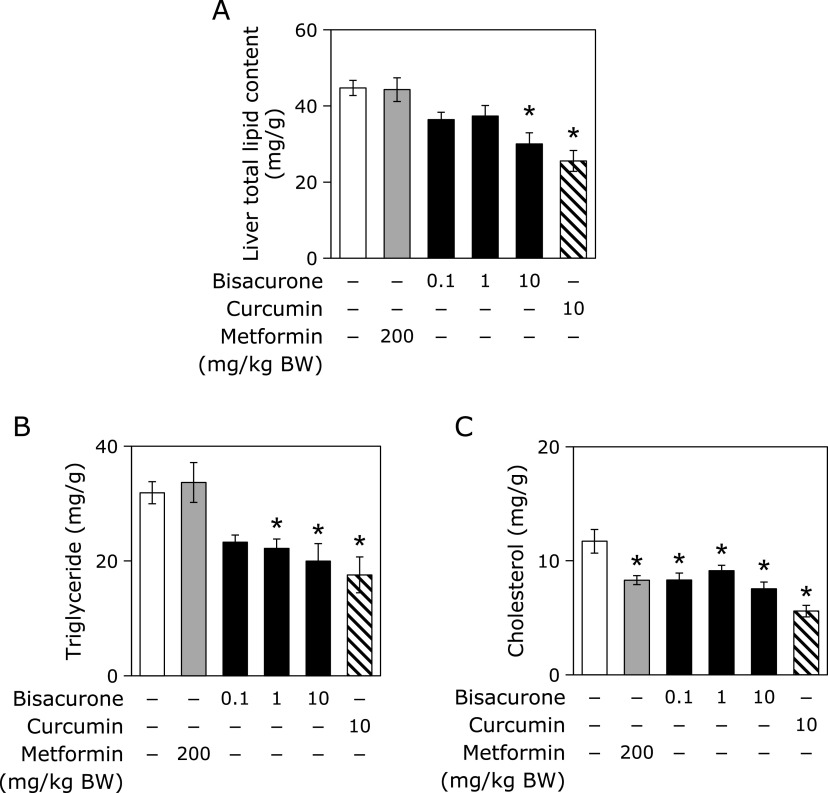
Effects of bisacurone on the hepatic lipid content in mice. Mice were administrated with 0.1, 1, or 10 mg/kg BW bisacurone and 10 mg/kg BW curcumin for 7 consecutive day. Metformin at 200 mg/kg BW and polyethylene glycol were used as the positive and negative controls, respectively. (A) Total lipids contents, and (B) triglyceride and (C) total cholesterol levels were measured as described in the Material and Methods section. Data are the mean ± SE (*n* = 6). Statistical analysis was performed using Dunnett’s test. *p<0.05 vs DMSO-treated control cells.

Although there was no change in the liver weight among all the groups (Table 1), oral administration of bisacurone decreased total lipid content at 10 mg/kg BW in the liver (Fig. 6). The level of triglyceride decreased in the groups of bisacurone at 1.0 and 10 mg/kg BW, and the level of total cholesterol also decreased in all bisacurone-dosed groups. The curcumin given group also decreased total lipid content, triglyceride and cholesterol levels in the liver. Interestingly, the metformin group showed a decrease in total cholesterol level in the liver, but there were no change in the total lipid content and triglyceride level. These results suggested that bisacurone and curcumin possible to reduce hepatic lipid storage without affecting plasma lipids.

### Effects of oral administration of bisacurone on lipogenesis in the liver of mice

**Fig. 7:**
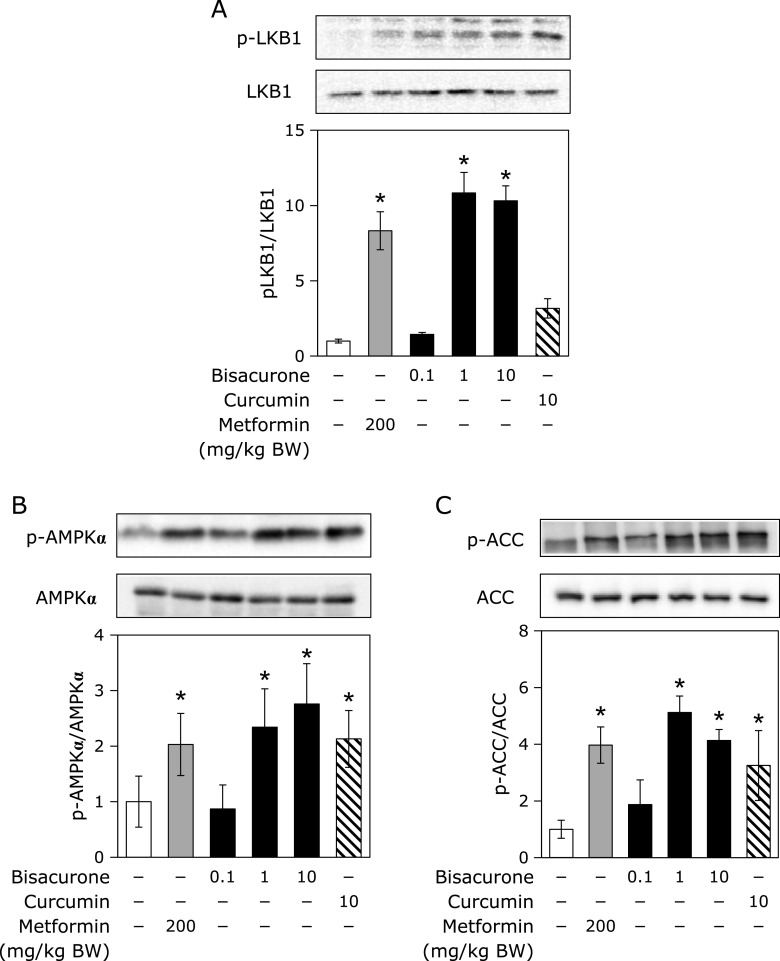
Effects of bisacurone on phosphorylation of LKB1, AMPKα, and ACCα in the liver of mice. Animal treatment is the same as shown in Fig. 6. The phosphorylation levels of LKB1, AMPKα, and ACCα, and their expression levels were determined by western blotting. A typical representative result was shown. Density of specific band for phosphorylated protein was normalized by that of corresponding protein expression. Data are the mean ± SE (*n* = 6). Statistical analysis was performed using Dunnett’s test. *p<0.05 vs DMSO-treated control cells.

**Fig. 8:**
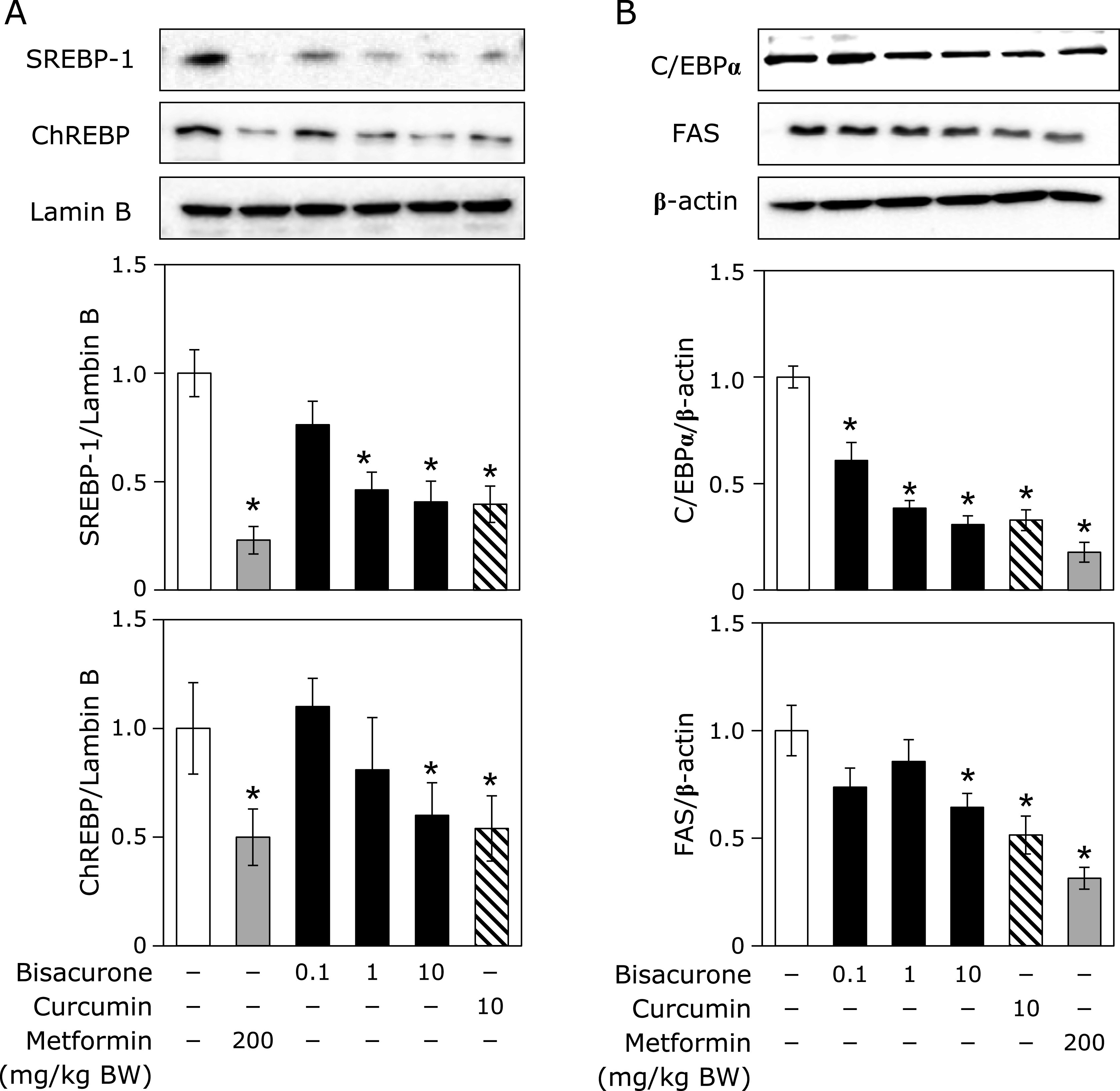
Effects of bisacurone on the lipogenesis-related factors in the liver of mice. Animal treatment is the same as shown in Fig. 6. (A) The nuclear translocation level of SREBP-1c and ChREBP and (B) the expression level of C/EBPα and FAS were determined by western blotting. Density of specific band was normalized by that of lamin B (for A) and β-actin (for B) expression. Data are the mean ± SE (*n* = 6). Statistical analysis was performed using Dunnett’s test. *p<0.05 vs DMSO-treated control cells.

Since AMPK modulates lipogenesis synthesis, lipolysis, and fatty acid oxidation, it was investigated that phosphorylation of AMPKα and its downstream lipogenesis enzyme ACC and upstream kinase LKB1. As shown in Fig. 7, administration of bisacurone at 1.0 and 10 mg/kg BW significantly increased the phosphorylation levels of AMPKα, ACCα, and LKB1 in the liver of mice. The similar results were observed in the curcumin- and metformin-given groups, except curcumin failed increased LKB1 phosphorylation. As the downstream events for the AMPK activation, the nuclear translocation levels of SREBP-1 and ChREBP in the liver were measured (Fig. 8A). Administration of bisacurone decreased the nuclear levels of SREBP-1 and ChREBP in a dose-dependent manner, and significant decreased was observed at 1.0 and 10 mg/kg BW. Curcumin and metformin also decreased them. On the other hand, these compounds did not affect the levels of SREBP-1 and ChREBP in the post-nuclear fraction (data not shown). Since FAS and C/EBPα are involved in hepatic lipid accumulation,^(12)^ their expression levels were further examined in this study. It was found that bisacurone significantly decreased the expression level of FAS and C/EBPα in the liver (Fig. 8B). Curcumin and metformin also decreased their expression levels. These results strongly suggest that bisacurone can reduced the lipogenesis in the liver not only through the regulation of phosphorylation of AMPKα/ACCα pathway but also through the decrease in nuclear translocation of the transcriptional factors, SREBP and ChREBP.

### Effects of oral administration of bisacurone on lipolysis in the liver of mice

As to the lipolysis factors, we investigated the expression of PPARα and CPT1 and found that Bisacurone at at 1.0 and 10 mg/kg BW significantly increased the expression level of PPARα and CPT1 in the liver. Curcumin, but not metformin, also showed the similar effects. It was reported that Fatty acid binding protein 4 (FABP4) is a target gene of PPARα.^(30)^ Therefore, we further investigated the expression level of FABP4. It was found that the expression level of FABP4 was similar to that of PPARα and CPT1, i.e., bisacrone and curcumin, but not metformin, increased the FABP4 expression.

## Discussion

**Fig. 9:**
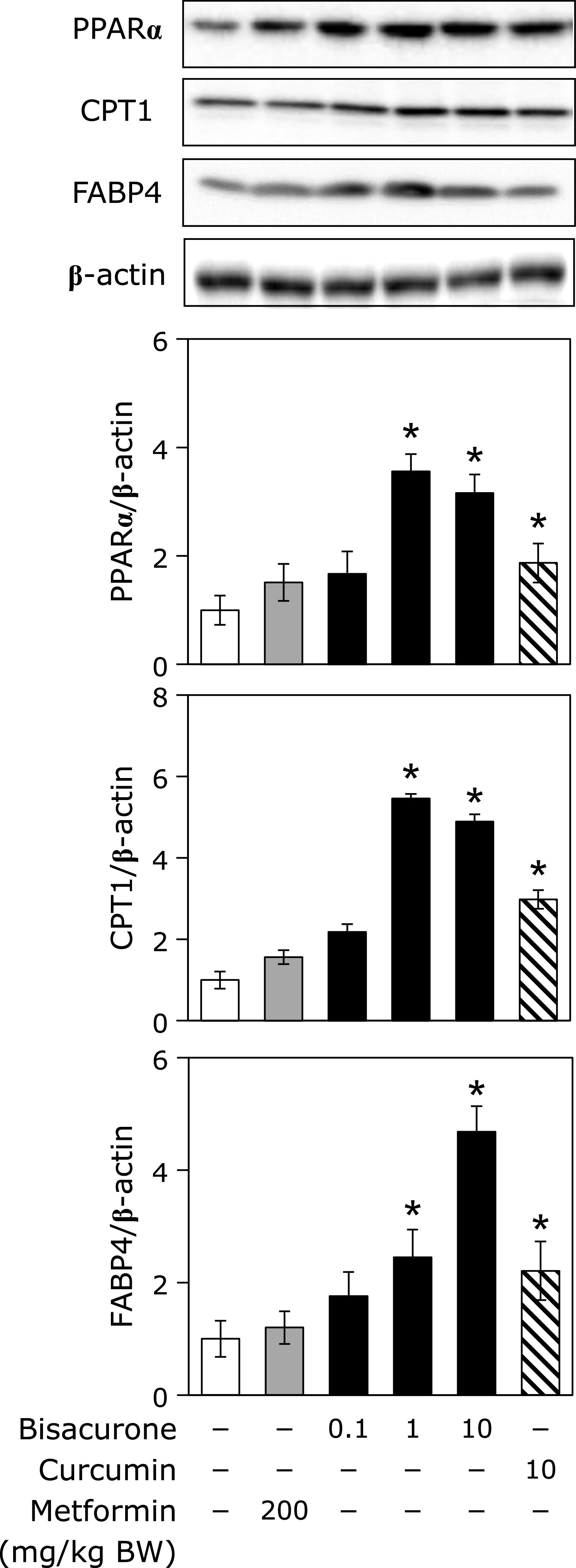
Effects of bisacurone on the lipolysis-related factors in the liver of mice. Animal treatment is the same as shown in Fig. 6. The expression levels of PPARα, CPT1, and FABP4 were determined by western blotting. Density of specific band was normalized by that of β-actin expression. Data are the mean ± SE (*n* = 6). Statistical analysis was performed using Dunnett’s test. *p<0.05 vs DMSO-treated control cells.

**Fig. 10:**
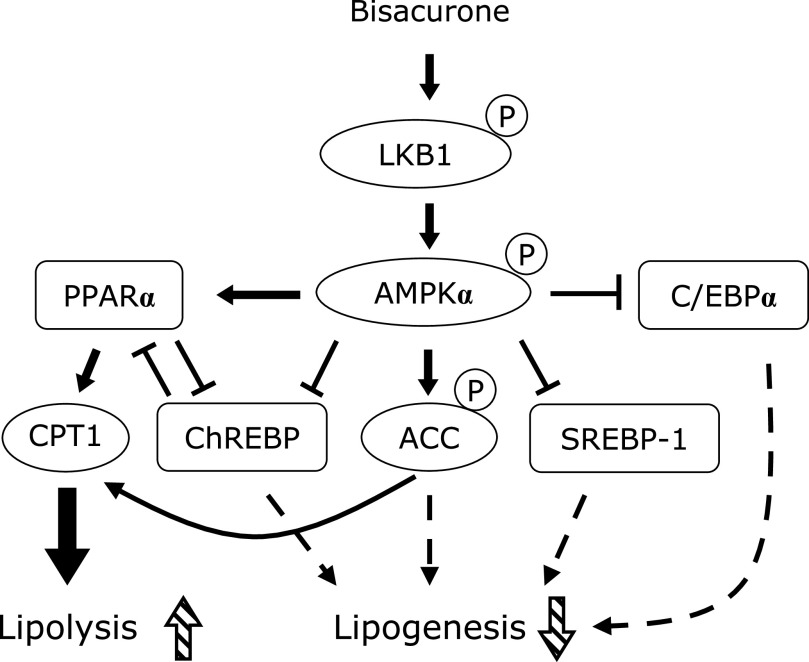
A speculated mechanism of bisacurone for the reduction of lipid accumulation in the liver.

In this study, we demonstrated that bisacurone, which is one of the sesquiterpenes in turmeric,^(20,21)^ prevented hepatic lipid accumulation in HepG2 cells and modulates lipid metabolism in the liver of ICR mice at the compound level. In HepG2 cells, bisacurone inhibited saturated fatty acids-induced lipid accumulation (Fig. 3) through AMPK-driven pathways, i.e., inhibiting lipogenesis through phosphorylation of ACC (Fig. 4) and promoting lipolysis and fatty acid oxidation through upregulation of PPARα and CPT1 (Fig. 5). In the liver of mice, bisacurone also inhibited lipogenesis through the nuclear translocation of SREBP-1 and ChREBP, and downregulation of C/EBPα and FAS (Fig. 8), in addition to phosphorylation of ACC by AMPK (Fig. 7). On the other hand, bisacurone enhanced lipolysis in the liver of mice, accompanied by increasing PPARα, CPT1, and FABP4 (Fig. 9). Taken these results together, a speculated mechanism is shown in Fig. 10. This is the first report that bisacurone attenuated hepatic lipids by modulating lipid metabolism.

It is known that curcumin, a major polyphenolic constituent of turmeric, has been reported to modulate lipid metabolism,^(31–33)^ but little is known about the health beneficial functions of bisacurone at the compound level. Only one report demonstrated that bisacurone inhibited adhesion of inflammatory monocytes and oral cancer cells to human umbilical vein endotherial cells through down-regulation of tumor necrosis factor α-activated vascular adhesion molecule-1,^(25)^ suggesting that bisacurone has the potential to suppress inflammation. Another sesquiterpene, ar-turmerone also suppressed inflammation by inhibiting tumor necrosis factor α-induced phosphorylation of IκBα and NFκB p65 subunit.^(21)^ Hot water extract of *Curcuma longa* L. (WEC), which is rich in sesquiterpenes including bisacurone and poor in curcuminoids, reveled the similar effect.^(34)^ Recently, WEC was reported to improve inflammation in subject with overweight or pre- or mild hypertension by a randomized, double-blind, placebo controlled trial.^(35)^ WEC was also reported to inhibit hepatic oxidative stress and inflammation, resulting in suppressing acute ethanol-induced liver injury in mice^(36)^ and non-alcoholic steatohepatitis.^(37)^ The former report demonstrated that WEC prevented the ethanol-increased formation of lipid droplets, though there is no quantified data, whereas the latter report showed that intake of WEC did not alter hepatic triglyceride and total cholesterol. Therefore, there is no consistency about the effects of WEC on hepatic lipid accumulation. Moreover, the active compound in WEC is unclear. However, from the results in these previous studies, a clue arises that bisacurone has a potency to prevent nonalcoholic fatty liver disease and can be an active compound in WEC. Although our findings clearly demonstrate that purified bisacurone decreased hepatic triglyceride and total cholesterol levels, there is a limitation of the results that decreasing hepatic lipid accumulation has been estimated in mice fed standard diet, but not high-fat diet. Further study is needed to clarify whether bisacurone also prevents fat accumulation in the liver of obesity model animals including high-fat diet-caused obese animals.

The underlying mechanism of bisacurone on decreasing hepatic lipids is mainly mediated by AMPK-driven pathways. Bisacurone decreased lipogenesis in saturated fatty acids-treated HepG2 cells and the liver of ICR mice accompanied by promoting phosphorylation of ACC, as a target of AMPK (Fig. 4 and 7). Furthermore, bisacurone decreased the nuclear translocation of SREBP-1 and ChREBP, and their target gene, FAS (Fig. 8). SREBP-1 is synthesized as precursor protein that is inserted in to the endoplasmic reticulum membrane. The precursor SREBP-1 undergoes proteolytic processing to release the transcriptionally active N-terminal domain, subsequently translocate into the nucleus and promotes its target genes.^(38)^ Previous study has demonstrated that the increased phosphorylation of AMPK promotes the phosphorylation of SREBP-1 (Ser372), which inhibits the cleavage and nuclear translocation of SREBP-1, thereby reducing transcriptional activity and the expression target genes.^(39)^ In addition to AMPK, PPARα also inhibits both the expression and cleavage of SREBP-1.^(40)^ Lee *et al.*^(41)^ reported that PPARα suppresses SREBP-1 processing through inducing its target, insulin induced gene 2a, which binds to SREBP-1 and prevents translocation of SREBP-1 to the Golgi apparatus during nutrient starvation in the liver. The activity of ChREBP is also reduced by both AMPK and PPARα.^(42,43)^ These results indicate that bisacurone decreased the expression of lipogenic proteins FAS and ACC through AMPK- and PPARα-mediated SREBP-1- and ChREBP-pathways in the liver. In addition to these pathways, bisacurone decreased the expression of C/EBPα that regulates lipogenesis in the liver. It is reported that C/EBPα knockout mice revealed significant reduction of hepatic triglyceride level by down-regulating liypogenic genes despite obesity.^(12)^ In 3T3-L1 cells, AMPK activator, AICAR, reduced the expression of C/EBPα,^(44)^ suggesting activation of AMPK downregulates the expression of C/EBPα. This report supports our findings that bisacurone activated AMPK pathway and decreased the expression of C/EBPα.

PPARα plays both inhibiting the lipogenesis and promoting the lipolysis in the liver. In the present study, we found that bisacurone increased PPARα at the protein expression level in both fatty acids-treated HepG2 cells (Fig. 5) and in the liver of ICR mice (Fig. 9). The expression and activity of PPARα are regulated various kinds of signaling pathways, including AMPK-pathway.^(13)^ Fasting-activated AMPK by a high AMP/ATP ratio leads to increase the PPARα activity in hepatocytes, while glucose-caused inactivation of AMPK suppresses the gene expression of PPARα in β-cells.^(45,46)^ On the other hand, the activity of PPARα is decreased by ChREBP.^(43)^ These results are in agreement with our findings that bisacurone increased the phosphorylation of AMPK and the expression of PPARα, and suppressed the nuclear translocation of ChREBP. The activity of CPT-1, the downstream target protein of PPARα, is regulated by AMPK.^(47)^ Phosphorylation of ACC by AMPK leads to inhibit the ACC activity and decrease the content of malonyl-CoA, resulting an increase in the CPT-1 activity through cancelling the inhibitory effect of malonyl-CoA against CPT-1.^(47,48)^ Taken together, these results suggest that bisacurone-promoted phosphorylation of AMPK is involved in both inhibiting lipogenesis and promoting lipolysis. Meanwhile, metformin did not affect the expression of PPAR and its targets, CPT1 and FABP4 in the liver (Fig. 9). In general, metformin-treated mice increase phosphorylation of AMPK and lipolysis in the liver, but several previous studies reported that short-term treatment of metformin did not affect the lipolysis in the liver of mice and cultured hepatocytes.^(49,50)^

Although both bisacurone and curcumin increased phosphorylation of AMPK and its substrate, ACC, bisacurone but not curcumin promoted phosphorylation of LKB1 as an upstream kinase (Fig. 7). This discrepancy suggest that the molecular target of bisacurone is different from that of curcumin. It is known that both LKB1 and calcium/calmodulin-dependent protein kinase kinase (CaMKK) are involved in AMPK activation. Previous study reported that curcumin analogue, dibenzoylmethane increased phosphorylation of AMPK and CaMKK inhibitor decreased dibenzoylmethane-induced phosphorylation in murine muscle cells.^(51)^ On the other hand, the some previous reports demonstrated that curcumin activates AMPK through LKB1.^(52,53)^ Thus, the upstream kinase for curcumin-caused phosphorylation of AMPK is the chaotic situation. It needs further study about the molecular target of bisacurone in future.

## Figures and Tables

**Table 1:** Changes in the body and tissue weight after administration of bisacurone, curcumin, and metformin for 7 days

	Control	Metformin (mg/kg BW)		Bisacurone (mg/kg BW)				Curcumin (mg/kg BW)
		200		0.1	1.0	10		10
Body weight (g)	27.76 ± 0.55	28.25 ± 0.94		28.63 ± 0.70	27.35 ± 0.80	28.03 ± 0.71		27.68 ± 0.46
Liver weight (% of body weight)	5.99 ± 0.28	6.27 ± 0.20		6.40 ± 0.13	6.61 ± 0.49	6.68 ± 0.18		6.37 ± 0.23
White adipose tissue weight (% of body weight)								
Epididymis	1.53 ± 0.09	1.61 ± 0.10		1.48 ± 0.07	1.22 ± 0.16	1.40 ± 0.16		1.25 ± 0.22
Perirenal	0.44 ± 0.03	0.42 ± 0.03		0.38 ± 0.02	0.32 ± 0.07	0.39 ± 0.06		0.33 ± 0.06
Mesenteric	0.91 ± 0.07	0.84 ± 0.13		0.74 ± 0.05	0.51 ± 0.10*	0.72 ± 0.09		0.65 ± 0.15
Subcutaneous	3.26 ± 0.25	3.18 ± 0.44		2.95 ± 0.17	2.44 ± 0.47	2.58 ± 0.56		2.46 ± 0.38
Total	6.13 ± 0.31	6.04 ± 0.65		5.54 ± 0.14	4.49 ± 0.75	5.09 ± 0.78		4.70 ± 0.71
Brown adipose tissue weight (% of body weight)	0.41 ± 0.04	0.38 ± 0.03		0.48 ± 0.08	0.39 ± 0.04	0.42 ± 0.05		0.40 ± 0.04

*Significant difference against control group (*p*<0.05) by Dunnett’s test. BW, body weight.

## Abbreviations

ACC: acetyl-CoA carboxylase
AMPK: AMP-activated protein kinase
BW: body weight
ChREBP: carbohydrate-responsive element-binding protein
C/EBP: CCAAT/enhancer binding protein
FABP4: fatty acid binding protein 4
FAS: fatty acid synthase
LKB1: liver kinase B1
PPAR: peroxisome proliferator-activated receptor
SREBP: sterol regulatory element-binding protein
WEC: water extract of *Curcuma longa* L.
